# Effect of antifibrinolytic drugs on transfusion requirement and blood loss during orthotopic liver transplantation: Results from a single center

**DOI:** 10.4103/0973-6247.42693

**Published:** 2008-07

**Authors:** Surekha Devi A., Dharmesh Kapoor, P. B. N. Gopal, M. Subrahmanyam, R. S. Ravichandra

**Affiliations:** *Department of Transfusion Medicine, Global Hospitals, Hyderabad, India*; 1 *Department of Hepatology, Anaesthesiology, Global Hospitals, Hyderabad, India*; 2 *Department of Critical Care, Global Hospitals, Hyderabad, India*

**Keywords:** Antifibrinolytics, blood transfusion, fibrinolysis, liver transplantation

## Abstract

**Background:**

During orthotopic liver transplantation (OLT), activation of the fibrinolytic system can contribute significantly to perioperative bleeding. Prophylactic administration of antifibrinolytic agents has been shown to reduce blood loss and the need for allogenic transfusion.

**Objective:**

To study the effect of antifibrinolytics on requirement of blood components, blood loss and operative time during OLT in patients with end stage liver disease, reporting to a single centre.

**Materials and Methods:**

Consecutive patients who underwent OLT at this centre during the period February 2003-October 2007 were the subjects of this study. Based on the individual anesthesiologist's preference, patients were assigned to receive either two million units of aprotinin (AP) as a bolus followed by 5,00,000 units/hour or 10 mg/kg tranexamic acid (TA) as a bolus followed by 10 mg/kg every six to eight hours, administered from the induction till the end of the surgery. Transfusion policy was standardized in all patients. Intraoperative red cell salvage was done wherever possible. The effect of these two antifibrinolytic drugs on transfusion requirement was evaluated as a whole and in a sub group of patients from each treatment group and compared with a concurrent control group that did not receive antifibrinolytic drugs.

**Results:**

Fifty patients (40 M / 10 F, 44 adults, 6 pediatric patients) underwent OLT in the study period. Fourteen patients were given AP, 25 patients were given TA and 11 patients did not receive any of the agents(control group). The median volume of total blood components transfused in antifibrinolytic group (n = 39) was 4540 ml(0-19,200ml), blood loss 5 l(0.7-35l) and operative time 9h (4.5-17h) and that of control group(n = 11) was 5700 ml(0-15,500ml), 10 l(0.6-25 l) and 9h (6.4-15.8h) respectively. The median volume of blood transfusions, blood loss and operative time was lesser in AP group(n = 14) than that of TA group(n = 25).

**Conclusion:**

There is definite decrease in transfusion requirement, blood loss and operative time in the patients who received antifibrinolytic drugs than that of patients who did not receive. Because of the small sample size, comparisons carried between different groups did not show statistical significance. Prophylactic use of antifibrinolytics during OLT, possibly helps in blood conservation.

## Introduction

Of all solid organ transplantations, orthotopic liver transplantation (OLT) has placed the greatest demands on clinical transfusion services.[1] OLT has become an accepted treatment for end-stage chronic liver disease, with one year patient survival rates of 80% to 90%.[2] OLT requires complex surgical dissections and suturing of major vascular structures which is responsible for surgical blood loss.[3] In addition to the procedure related to surgery, abnormal bleeding typically occurs during liver transplantation as a consequence of severe hemostatic dysfunction[4] Etiology of hemostasis abnormalities is multifactorial, including deficit in platelets and coagulation factors related to existing liver disease and increased fibrinolysis, which can contribute significantly to nonsurgical blood loss.[4] Pathological activation of the fibrinolytic system is related to the presence of huge amounts of circulating tissue type plasminogen activator (t-PA) as a result of lack of tissue plasminogen activator (t-PA) clearance during the anhepatic phase and a burst release of t-PA associated with the reperfusion of the ischemic graft.[4] The t-PA converts plasminogen into plasmin. Plasmin degrades fibrin leading to the premature breakdown of hemostatic clots and subsequent increased blood loss and transfusion requirements.[2] Kang reported that 82.5% of patients showed signs of hyperfibrinolytic activity in at least one blood sample during OLT.[5] Identification of hyperfibrinolysis as one of the underlying mechanisms of increased blood loss during liver transplantation has provided support for a more goal-directed therapy, using antifibrinolytic drugs.[2] Antifibrinolytic drugs are available as direct inhibitors of plasminogen (lysine analogs eg. Tranaxemic acid) or as inhibitors of plasmin (serine protease inhibitors e.g. Aprotinin).[2] The associated coagulopathy, anemia, malnutrition and severe portal hypertension have made this procedure more daunting and the use of blood products almost universal.[6] In the early 90s, Mor *et al*, from Dallas, were among the first to report on the negative association between intraoperative blood transfusion requirement and postoperative outcome variables, such as graft and patient survival, length of the stay in the intensive care unit, and infectious complications.[2] A number of prospective, placebo-controlled trials have shown the efficacy of prophylactic antifibrinolytic agents in reducing blood loss and blood transfusion requirements during OLT.[4] There is no consensus on how, when, or which antifibrinolytic should be used. As antifibrinolytic therapy will induce a shift of the hemostatic balance toward coagulation, thrombotic complications may be feared at the outset in patients with thrombotic risk factors. This consideration supports the view that the decision to use antifibrinolytics should be taken on an individual basis and that it should not be routine.[7]

**Figure 1:**
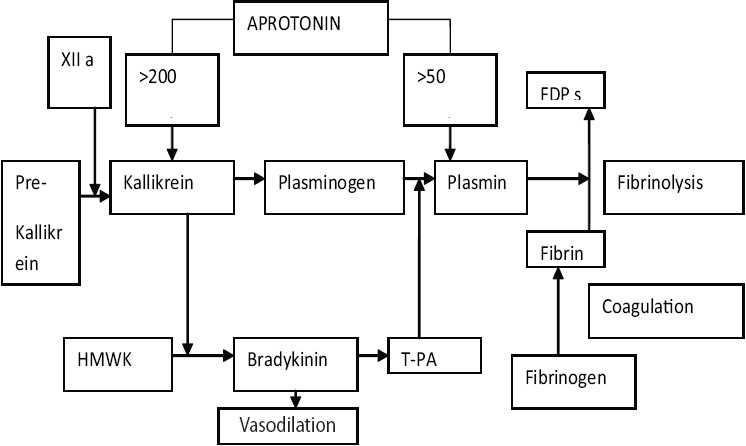
Schematic presentation of the influence of aprotonin on fibrinolysis and the kallikrein-kinin pathway. Aprotonin is known as an inhibitor for plasmin (at concentrations of >50 KIU/mL) and kallikrein (at concentrations of > 200 KIU/mL). Plasmin inhibition leads to a reduction of fibrinolysis. Inhibition of the kallikreinkinin pathway leads to a reduction of bradykinin formation, which may explain the improved hemodynamic stability after graft reperfusion, but it also decreases the formation of t-PA.

AP is obtained from bovine lung tissue, and its administration may be associated with anaphylactic reaction after repeated exposure.[4] Thromboembolic events have been reported after AP administration.[4] There is risk of prion transmission as it is derived from bovine tissue.[4] TA results in few allergic reactions and is not associated with a risk of disease transmission. TA is far less expensive than AP. Since they have similar hemostatic effects, TA is preferred over AP as an antifibrinolytic agent in OLT.[4] The influence of AP on fibrinolysis is shown in schematic representation as shown in Figure 1.

The primary end point was to study the effect of anti fibrinolytics on requirement of blood components, blood loss and operative time during OLT in patients with end stage liver disease (ESLD) reporting to our center and compare the same parameters with a control group who did not receive these drugs. We also compared transfusion requirement, blood loss and operative time in the cohort which received AP vis-à-vis TA.

## Materials and Methods

Consecutive patients who underwent OLT at this center during the period February 2003 - October 2007 were the subjects of this study. Exclusion criteria included previous exposure to AP and portal vein thrombosis.

Based on the individual anesthesiologist's preference, patients were randomly assigned to receive either AP or TA. AP (Aprostat 500,000 KIU in 50 ml, Samarth Pharma Pvt. Ltd. Mumbai, India) was given in a dose of two million KIU as a slow bolus after test dose during induction followed by 500,000 KIU/hour throughout the surgery. In children, the dose was 10,000 KIU/kg as a slow bolus after test dose followed by 5,000 KIU/hour throughout the surgery. TA (Tranemic acid 500mg in 5 ml, Windlas Biotech Ltd. Dehradun, India) was given in a dose of 10 mg/kg as a slow bolus during induction followed by 10 mg/kg every 6-8 hours, till the end of the surgery in 10 patients. In remaining 15 patients it was given as 10 mg/kg as bolus during induction followed by 5 mg/kg/hr as infusion till early reperfusion phase. General anesthesia was standardized. Anesthetic management was performed using a common protocol previously decided by consensus by the anesthesia team. Rapid sequence induction was done with propofol and suxamethonium. Atracurium was used for muscle relaxation. Direct arterial pressure and central venous pressure were monitored in all cases. All patients were equipped with radial and femoral arterial catheters. Pulmonary artery catheters were used in high risk cases.(Child C cirrhosis, pulmonary hypertension and cardiac failure) All patients were warmed with warming blankets to obtain normothermia. Regular laboratory monitoring of arterial blood gases, (Ciba Corning 644, USA) serum electrolytes, (Beckman coulter, USA) lactate, (end point method, Randox kit, Randox laboratories Ltd. UK BT 29 4QY) hemoglobin and platelet count, (HMX Beckman coulter, 5 part differential, USA) prothrombin time (Clotting method, Thromborel S, Dade Behring GmbH, Germany) and fibrinogen levels (Clauss method using Fibroquant, Tulip Diagnostics (P) Ltd, Goa, India) are done. A balanced anesthetic technique was used for maintenance of anesthesia.

Transplantation was performed with piggyback technique (retrohepatic caval vein was preserved) in both living donor liver transplantation (LDLT) and DDLT. Venovenous bypass was not used in any of the patients. After hepatectomy, the allograft was implanted in orthotopic location. Sequential anastomosis of vena cava, portal vein and hepatic artery of the donor to recipient was done. After the blood flow was restored to the new liver, biliary anastomosis was constructed to the recipient's bile duct. Surgical hemostasis was achieved by electrocautery, argon beam coagulation, usage of fibrin glue and end to side porto-caval shunt wherever indicated. The allografts were preserved in University of Wisconsin (UW) solution (Bristol-Myers Squibb Co., USA) or Histidine-tryptophan-ketoglutarate (HTK) solution (HTK Custodial, Dr.Frank Kohler Chemie GmbH, Germany). Transfusion policy was standardized in all patients. Blood products were screened for anti-human immunodeficiency virus (HIV) 1and2, hepatitis B surface antigen (HbsAg), anti-hepatitis C virus (HCV) and anti-hepatitis B core (HBc) antibodies by enhanced chemiluminescence technique (Vitros EciQ, Ortho-Clinical Diagnostics, USA). Cellular blood products were also screened for anti-cytomegalo virus (CMV) IgM antibodies by Elisa method (Human, Gesellschaft fur Biochemica und Diagnostica mbH, Weisbaden, Germany) irrespective of CMV status of the recipient. Leucoreduction of cellular blood products was done using third generation laboratory WBC filters (Immugard III-RC and PL, Terumo Corporation, Japan). Criteria for replacement of blood products were as follows:

Administration of red cell units (red cells were procured in saline adenine glucose-mannitol) to maintain hemoglobin (Hb) levels at 8 gm%; fresh frozen plasma (FFP) in case of hemorrhage associated with International Normalized Ratio (INR) >2.0; apheresed platelets to maintain platelet count >50×10^3^/mm^3^ and cryoprecipitate for fibrinogen levels<100 mg/dl in the presence of ongoing bleeding. In minor ABO mismatched transplants (eg, group O allografts transplanted to nongroup-O patients, nongroup-AB allografts transplanted to group AB patients), the following transfusion protocol was followed: transfusing recipient group red cell units at the beginning of surgery, but switching to donor group red cell units for the final part of surgery i.e. after the graft is implanted and for subsequent postoperative transfusions. Plasma products were transfused based on recipient ABO group. After six weeks, if direct antiglobulin (DAT) and anti-A/anti-B antibodies were negative, red cells were switched over to recipient group.[9] Data are provided in units. The total volume of blood products given to every patient during intraoperative period was calculated. When transfusions exceeded more than one blood volume within 24h, it was considered as massive transfusion.[9] All intravenous fluids and blood products except platelets were administered through blood warmer.

Intraoperative autotransfusion was done by using cell saver (C.A.T.S Fresenius, Germany). Unfractionated Heparin (Gland pharma Ltd. Hyderabad) in a dose of 1 unit / 1 ml of saline flush was used as anticoagulant for salvaged blood. Red cell salvage was not done in the presence of hepatocellular carcinoma (HCC), infection, and when blood loss was < 1500 ml. Intraoperative blood loss was quantitated by weighing of sponges and pads, and measuring the volume of blood collected in suction containers. Thromboembolic complications within one week were reported. Hepatic artery and portal vein thrombosis were diagnosed by screening the patients with color pulsed doppler sonography and if there was any sonographic evidence, confirmed on direct angiography.

### Statistical analysis

SPSS windows version 15.0 was used for statistical analysis. Data are expressed as median and range. A ‘p’ value of less than 0.05 was considered significant.

### Results

Out of 50 patients who underwent orthotopic liver transplantation (OLT), 44 were adults and six were pediatric patients. The median age at the time of transplantation was 46 years (range: 4 to 67 yrs). There were 40 male recipients and 10 female recipients. Out of 50 patients, 39 patients underwent deceased donor liver transplantations (DDLT). Of the 39, 37 patients received whole liver allograft, while two patients shared split graft (adult received right lobe graft and child received left lateral segment). Eleven patients underwent living donor liver transplantations (LDLT); while seven were adult to adult, four were adult to child. Of 50, 35 patients received graft from identical blood group organ donors; while 15 patients received graft from non-identical blood group organ donors (minor ABO mismatched transplants). Out of 50 patients, 12 patients had alcoholic cirrhosis, 15 patients had post viral cirrhosis, 14 patients had cryptogenic cirrhosis and nine had cirrhosis due to other causes. Depending on the intensity of liver disease, Child-Turcotte-Pugh score was given. There was one patient in Child A category, 29 patients in Child B category and 20 in Child C category. Out of 50 patients, allograft was preserved in HTK solution for six patients and in UW solution for 44 patients. Out of 50, 39 patients were given prophylactic antifibrinolytics (AP was given to 14 patients, tranexamic acid (TA) was given to 25 patients) and 11 patients were included in the control group and did not receive antifibrinolytics. The success of any transplant program depends on the survival rate. Of 50, 35(70%) patients are surviving.

Transfusion data and perioperative variables of antifibrinolytic group vs control group is shown in Table 1. Perioperative data of AP group vs TA group vs control group is represented in Table 2. There was decrease in the total volume of intraoperative transfusion requirement (median: 4500 ml vs 5700 ml; *P*=0.33), individual blood component requirement (Packed RBC median: 7 units vs 9 units, *P*=0.37; FFP median: 8 units vs 10 units, *P*=0.40; Cryo median: 20 units vs 10 units, *P*=0.71; SDP median: 1 unit vs 2 units, *P*=0.13), red cell salvage(median: 700 ml vs 1000 ml, *P*=0.59), operative time(median: 9h vs 9h, *P*=0.47) and blood loss(median: 5 lts vs 10 lts, *P*=0.09) in the antifibrinolytic group than that of control group. Within individual antifibrinolytic drug group (AP and TA) transfusion requirement(median: 3950 ml vs 4840 ml, *P*=0.39), individual blood component requirement(Packed RBC median: 6 units vs 8 units, *P*=0.32; FFP median: 8.5 units vs 8 units, *P*=0.99; Cryo median: 9 units vs 30 units, *P*=0.001; SDP median: 0 vs 1 unit, *P*=0.067), red cell salvage(median: 500 ml vs 837.5 ml, *P*=0.22), operative time (median: 8.83h vs 9h, *P*=0.41) and blood loss(median: 4.25 lts vs 5.8 lts, *P*=0.35) in AP group was less than that of TA group. Because of the small sample size of the patients, the comparisons carried out between different groups failed to show statistical significance though there was numerical difference.

**Table 1:** Perioperative data of the antifibrinolytic group vs control group (n=50)

Variable	Antifibrinolytic group (AP+TA; n=39)	Control (n=11)	*P* value
Total vol. transfusions (ml)	4540(0-19,200)	5700(0-15,500)	0.33
Packed RBC (units)	7(0-34)	9(0-20)	0.37
FFP (units)	8(0-30)	10(0-45)	0.40
Cryo (units)	20(0-60)	10(0-50)	0.71
SDP (units)	1(0-4)	2(0-5)	0.13
Auto-transfusion (intra-op red cell Salvage in ml)	700(90-1975)	1000(120-3450)	0.59
Operative time (hours)	9(4.5-17)	9(6.4-15.8)	0.47
Blood loss (litres)	5(0.7-35)	10(0.6-25)	0.09
ICU stay (days)	6(1-32)	6(3-22)	0.86
Hospital stay (days)	20(1-38)	21(17-37)	0.14

**Table 2:** Perioperative data of the subsets from the treatment groups vs control group

Variable	AP (n=14)	TA (n=25)	Control (n=11)	*P* value
Total vol. transfusions (ml)	3950(0-11,220)	4840(1000-19,200)	5700(0-15,500)	0.39
Packed red cells (units)	6(0-22)	8(1-34)	9(0-20)	0.32
FFP (units)	8.5(0-20)	8(2-30)	10(0-45)	0.99
Cryo (units)	9(0-30)	30(0-60)	10(0-50)	0.001
SDP (units)	0(0-3)	1(0-4)	2(0-5)	0.06
Auto-transfusion (intra-op red cell salvage in ml)	500(300-800)	837.5(90-1975)	1000(120-3450)	0.22
Operative time (hours)	8.83(4.5-17)	9(5.5-17)	9(6.4-15.8)	0.41
Blood loss (liters)	4.25(0.7-11)	5.8(1.2-35)	10(0.6-25)	0.35
ICU stay (days)	7(4-15)	5.5(1-32)	6(3-22)	0.16
Hospital stay (days)	24(11-38)	19.5(1-32)	21(17-37)	0.006

Total intensive care unit (ICU) (median: 6 days vs 6 days, *P*=0.86) and hospital lengths of stay (median: 20 days vs 21 days, *P*=0.14) were not different between the antifibrinolytic group vs control group. None of the patients in the antifibrinolytic group developed thromboembolic phenomenon.

## Discussion

Prophylactic inhibition of hyperfibrinolysis with the biological serine protease inhibitor AP or the synthetic lysine analog TA is common clinical practice in OLT.[4] This study did show difference in blood loss, transfusion requirement, and operative time (lesser in antifibrinolytic group than that of control group), though there was no statistical significance in the two groups. This could possibly be due to the small sample size. Preoperative correction of coagulation defects has not been shown to be effective in reducing intraoperative bleeding. Throughout the procedure, a rapid and sensitive method for monitoring coagulation is necessary in order to guide the rational use of blood components and pharmacological agents.[10] Along with antifibrinolytics, we also gave cryoprecipitate transfusions during early reperfusion phase to combat fibrinolysis, guided by the plasma fibrinogen levels.

In minor mismatched liver transplants, donor passenger lymphocytes are transferred with the allograft at the time of transplantation and can produce donor derived antibodies(DDAb) in recipient.[9] Ramsey reported that the frequency of DDAb and hemolysis was 40% and 29% respectively in liver transplant recipients.[8] The ABO antibodies are typically IgG, have appeared 7 to 10 days after allotransplantation, and have persisted for a median 2 to 3 weeks.[8] This is heralded by the development of positive direct antiglobulin test. Although the hemolysis is usually mild and self-limited, substantial morbidity associated with hemolysis has been reported.[9] The frequency of passenger-lymphocyte-induced hemolysis is increased in patients taking cyclosporine or tacrolimus compared with earlier immunosuppressive treatments.[8] In order to prevent hemolysis, transfused red cells should be of organ donor ABO group to replace susceptible red cells with cells that will not be hemolyzed, plasma products should be of recipient ABO group to reduce the risk of hemolysis by providing soluble ABO antigen capable of neutralizing DDAb[9]. None of our patients who received allograft from non-identical blood group organ donors (n=15) developed passenger-lymphocyte-induced hemolysis as we followed the above said transfusion protocol for them.

The European Multicentre Study of Aprotinin in Liver Transplant (EMSALT) showed decrease in red blood cell usage with both high dose (2 × 10^6^ kalliekrin international unit (KIU) loading dose followed by 1 × 10^6^ KIU/h) and regular dose (2 × 10^6^ KIU loading dose followed by 0.5 × 10^6^ KIU/h) of aprotinin compared with placebo.[11] Due to its wide acting serine-protease inhibiting activity, aprotinin also has an effect on platelet function, and it has several anti-inflammatory properties.[12] Aprotinin use resulted in greater hemodynamic stability on reperfusion of liver graft, probably via inhibition of the kallikrein-bradykinin pathway.[2] Apart from reducing transfusion requirements in liver transplantation, it also reduced the need for intraoperative epinephrine, particularly at the time of reperfusion.[13] Even in this study, those who received aprotinin had lesser blood loss and received lesser volume of blood transfusions. A regular-dose aprotinin infusion has been reported to reduce both blood loss and the requirement for blood transfusion in patients undergoing liver transplantation and to be free of complications related to hypercoagulation.[14] However, many case reports have associated thrombosis and thromboembolism with antifibrinolytic administration during liver transplantation and open-heart surgery.[14] The anticoagulant, as opposed to a procoagulant effect of aprotinin has been demonstrated in patients undergoing liver transplantation. Clearly, a controversy exists regarding the safety of routine aprotinin use in liver transplant surgery.[14] Therefore, it is difficult to conclude from case reports alone whether there is a cause-effect relationship between the use of aprotinin and the occurrence of thromboembolic complications.

Aprotinin may reduce blood loss due to hemostatic defects, it is unlikely to have an effect on the amount of blood loss due to "hemostasis unrelated" factors.[15] In addition to enhanced fibrinolysis during OLT, many other causes lead to blood loss: coagulopathy, thrombocytopenia, thrombocytopathy, dysfibrinogenemia, dilutional coagulopathy, hypothermia, bleeding secondary to technical difficulties and variation in surgical experience and expertise.[16] Other side-effects that have been associated with the use of aprotinin are renal dysfunction and allergic reactions.[15] The advantages of antifibrinolytics counterbalance largely the disadvantages of these agents.

Dalmau *et al*, compared the efficacy of TA and AP in a double blinded, prospective, and randomized study.[16] Of 127 consecutive patients undergoing OLT, 64 patients received TA(10 mg/kg/h) and 63 patients received AP(2 million KIU bolus followed by 500,000 KIU/h infusion). There was no significant differences in coagulation test results or transfusion requirements of red cells, FFP, or platelets between two groups.[16] In this cohort, AP group had less blood loss and received lesser volume of blood transfusions than that of TA group, though there was no statistical significance.

Large transfusion requirements have been correlated with reduced graft survival, increased number of septic episodes, prolonged ICU and hospital stay, immune-suppression, immunomodulation, deterioration of liver regeneration, morbidity and mortality in patients undergoing OLT.[17] Blood conservation is not an "act" but a "science". Unfortunately, this has not been considered a priority for physicians and surgeons because deeply rooted concepts about the safety; ubiquitous availability and effectiveness of blood have not been challenged until recently.[6] In considering blood as an independent organ system, one must focus on the opportunity to optimize it.[6] Intraoperative red blood cell salvage and autologous transfusion is cost-effective in adult liver transplantation. Currently, where optimum resource utilization and fiscal constraint are paramount in healthcare delivery, autologous transfusion is an important adjunct in liver transplantation.[18] In our center, intraoperative red cell salvage was done during OLT wherever indicated, which was cost-effective for patients. Salvaged blood does not require screening for transfusion transmitted diseases and compatibility tests, wheras allogenic blood requires the same which costs the patient.

AP and TA both reduced transfusion requirements compared with controls in the study done on 1407 patients by Molenaar *et al*.[19] AP but not TA also significantly reduces intraoperative use of FFP.[18] Even in our cohort, red cell, FFP and other blood component requirement was less in AP group than that of TA group. In patients who received AP or TA, the overall perioperative incidence of venous thromboembolic events was comparable (1.4% and 0.7%, respectively).[19] In this study, neither venous embolism nor arterial thrombosis was observed in either group. Aprotinin has been studied more extensively in clinical trials and appears to offer more advantages compared to TA and Epsilon-Aminocaproic Acid (EACA).[16] The transfusion of red cells was 37% lower in high dose group and 20% lower in regular dose group who received AP.[16] Even in this cohort, Aprotinin group received lesser transfusion volume than that of TA group. However, because of the diverse population of liver transplant patients, the complicated coexisting medical conditions, the complex derangements of coagulation and fibrinolysis, and the potential adverse effects of antifibrinolytics, careful patient selection and close monitoring of patients receiving antifibrinolytics is prudent. The limitation of our study is the small sample size. Also, we have used subjectively determined objective transfusion triggers rather than utilizing objective parameters, as are delivered by the use of Thromboelastogram (TEG).

In conclusion, this study confirms the blood loss and blood transfusion reducing effect of AP and TA in patients undergoing liver transplantation and does not provide evidence for an increased risk of thromboembolic complications in liver transplant recipients who receive antifibrinolytic drugs. A larger study with bigger sample size is essential to establish significant reduction in blood loss and blood transfusion with antifibrinolytics.

Transfusion is associated with many risks and complications, and efforts to minimize blood loss and transfusion volume by using transfusion alternatives is recommended. Goal should be to develop strategies towards transfusion free environment.
